# Supplementary material for: Mechanism of glycogen synthase inactivation and interaction with glycogenin
Source: Nat Commun. 2022 Jun 11;13:3372. doi: 10.1038/s41467-022-31109-6 (PMC9188544; doi:10.1038/s41467-022-31109-6)
Supplement: Supplementary file 1 — Supplementary Information [file 41467_2022_31109_MOESM1_ESM.pdf]

Supplementary Information

Mechanism of glycogen synthase inactivation and interaction with glycogenin

Laura Marr [a], Dipsikha Biswas [b], Leonard A. Daly [c], Christopher Browning [d], Sarah Vial [d], Daniel P. Maskell [a], Catherine Hudson [d], Jay Bertrand [d], John Pollard [d], Neil A. Ranson [a], Heena Khatter [d], Claire E. Eyers [c], Kei Sakamoto [b], Elton Zeqiraj [a*]

[a] Astbury Centre for Structural Molecular Biology, School of Molecular and Cellular Biology, Faculty of Biological Sciences, University of Leeds, Leeds, UK

[b] Novo Nordisk Foundation Center for Basic Metabolic Research, University of Copenhagen, Copenhagen, Denmark

[c] Department of Biochemistry and Systems Biology, Institute of Systems, Molecular and Integrative Biology, University of Liverpool, Liverpool, UK

[d] Vertex Pharmaceuticals Inc., Milton Park, Abingdon, UK

*Correspondence to: e.zeqiraj@leeds.ac.uk

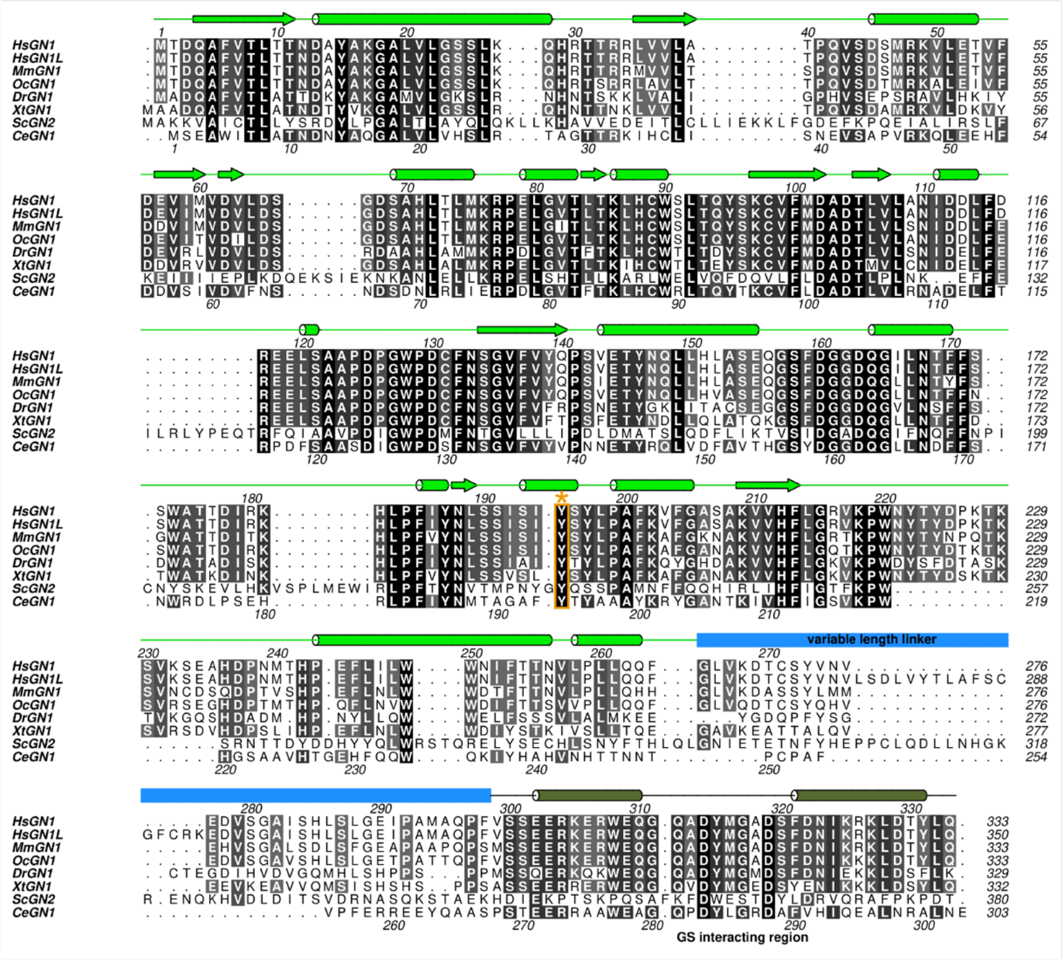

Supplementary Fig. 1 Sequence conservation of glycogenin

Multiple sequence alignment of GN from the indicated species (black = conserved, white = not conserved). Sequence alignment was performed with MUSCLE and edited and displayed using ALINE. The predicted secondary structure for HsGN is shown at the top of the alignment and coloured in green (analysed by PSIPRED). The human and *C. elegans* GN sequences are numbered at the top and bottom of the alignment respectively. The variable length linker is coloured in blue, the C-terminal GS interaction region is coloured in dark green and the tyrosine that is auto-glucosylated (Y195 in human) is boxed in orange and labelled with an asterix. *Hs, H. sapiens; Sc, S. cerevisiae; Ce C. elegans; Mm, M. musculus; Xt, X. tropicalis; Oc, O. cuniculus; Dr, D. rerio.*

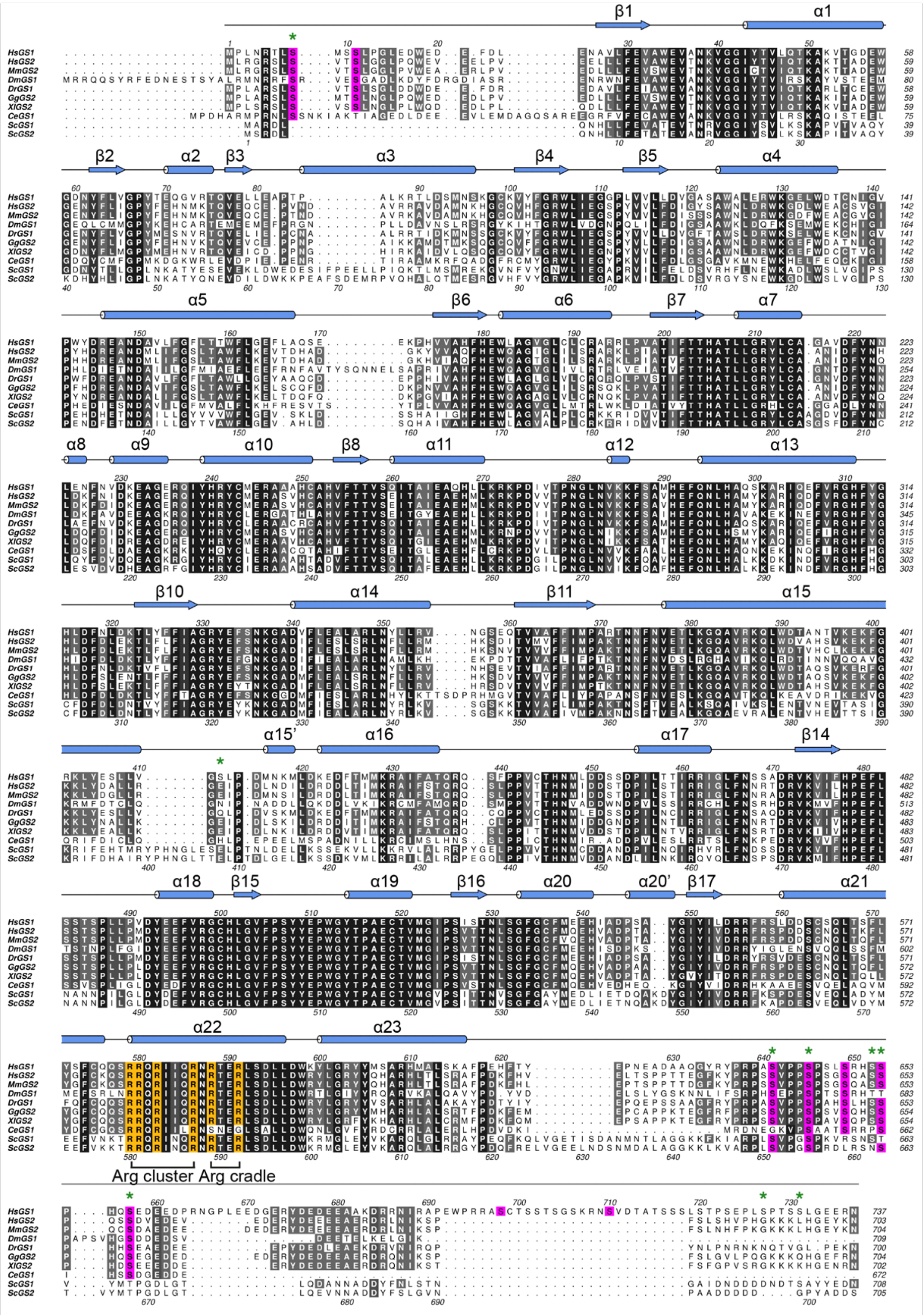

Supplementary Fig. 2 Sequence conservation of glycogen synthase

Multiple sequence alignment of GS from the indicated species (black = conserved, white = not conserved). Sequence alignment was performed with MUSCLE and edited and displayed using ALINE. The secondary structure for HsGS is shown at the top of the alignment and coloured in blue. The human and yeast GS sequences are numbered at the top and bottom of the alignment respectively. Known *in vivo* phosphorylation sites are shown in magenta. Residues that form the arginine cluster and arginine cradle are coloured in orange and labelled, and the phosphorylated residues identified by phosphomapping experiments in this study are shown by green stars. *Hs, H. sapiens; Sc, S. cerevisiae; Ce C. elegans; Dm, D. melanogaster; Mm, M. musculus; Xl, X. laevis; Gg, G. gallus; Dr, D. rerio.*

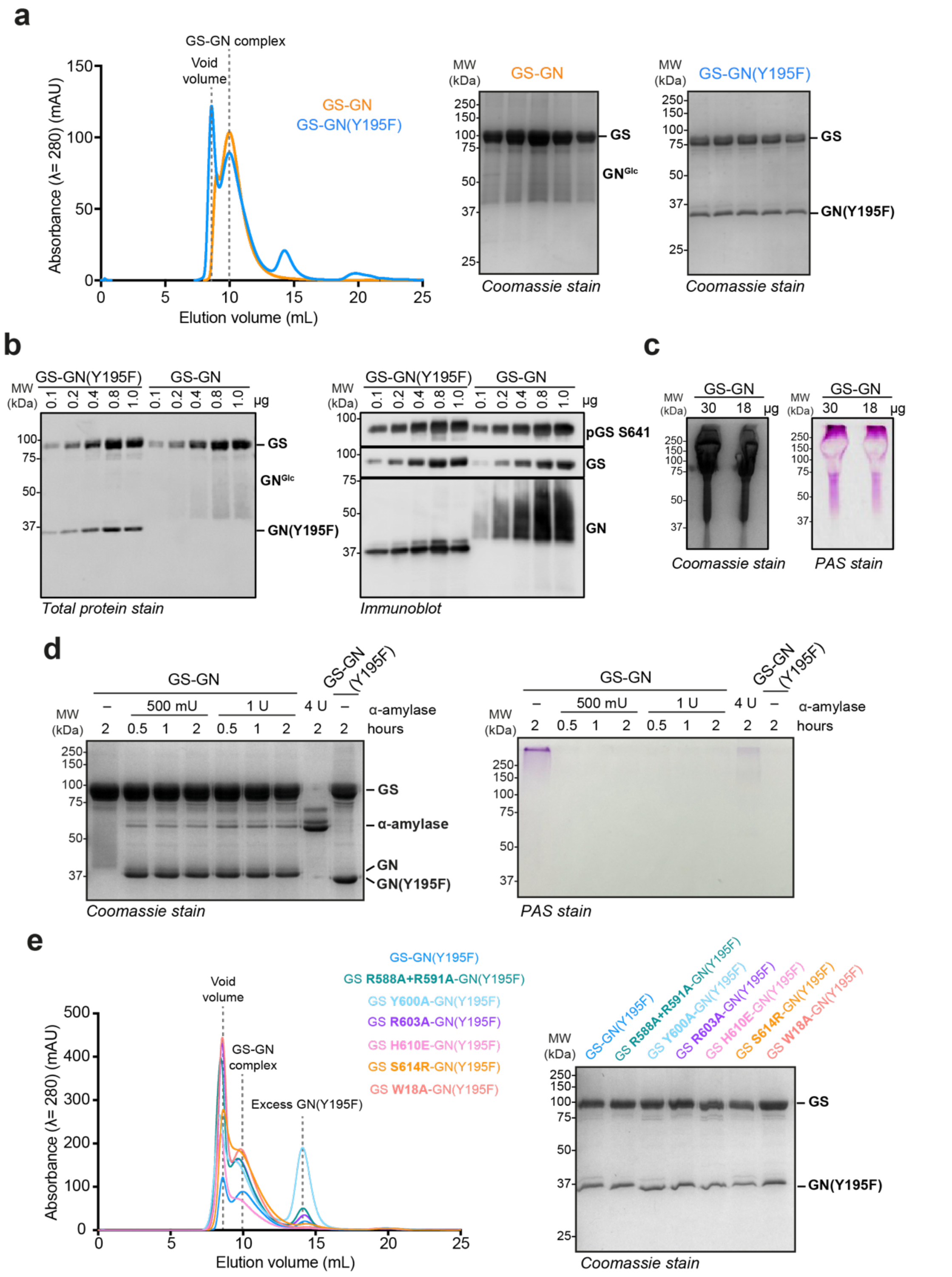

Supplementary Fig. 3 Complex purifications and analysis

a Size-exclusion chromatography profile of GS-GN Y195F (blue) and GS-GN WT (orange) (left), and SDS-PAGE analysis of the complex peak fractions (right). **b** Total protein stain and Immunoblot of GS-GN and GS-GN(Y195F) complexes. Data is representative of two independent experiments carried out in technical duplicates. **c** Coomassie and PAS stain of GS-GN to show sugars associated with the WT complex in the smeared band. Data is representative of two independent experiments. **d** Disappearance of the smeared band due to glucosylated GN, and appearance of single band for GN upon α-amylase treatment. Data is representative of three independent experiments. **e** Size-exclusion chromatography profile of GS WT and mutants in the GS-GN Y195F complex (left), and SDS-PAGE analysis of the corresponding mutants (right).

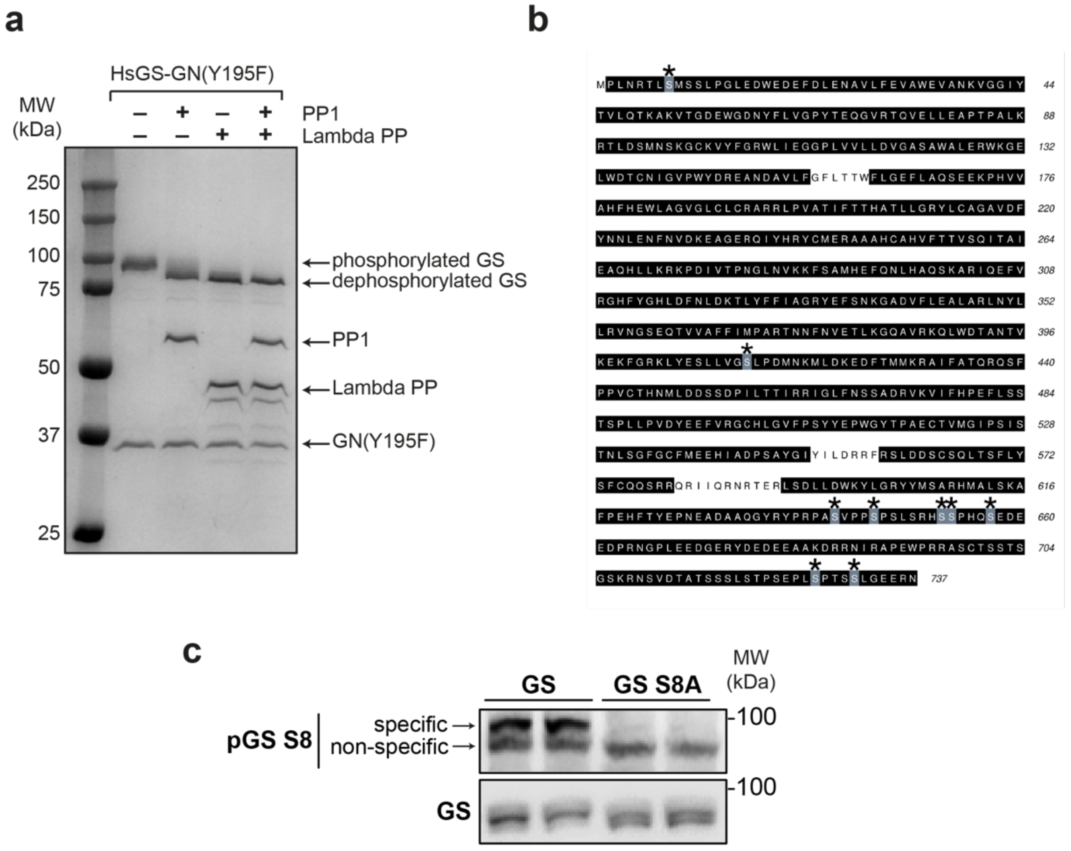

Supplementary Fig. 4 Human GS produced in insect cells is phosphorylated
a Shift in Human (Hs) GS band in SDS-PAGE to a lower molecular weight upon dephosphorylation by protein phosphatase 1 (PP1) and lambda protein phosphatase (lambda PP). Data is representative of three independent experiments. **b** Sequence of human GS. Sequence coverage (96.74%) during phosphorylation site mapping is highlighted. Identified phosphorylated residues identified are highlighted in grey and annotated with an asterix. **c** Validation of the pGS S8 antibody. Data is representative of two independent experiments carried out in technical duplicates.

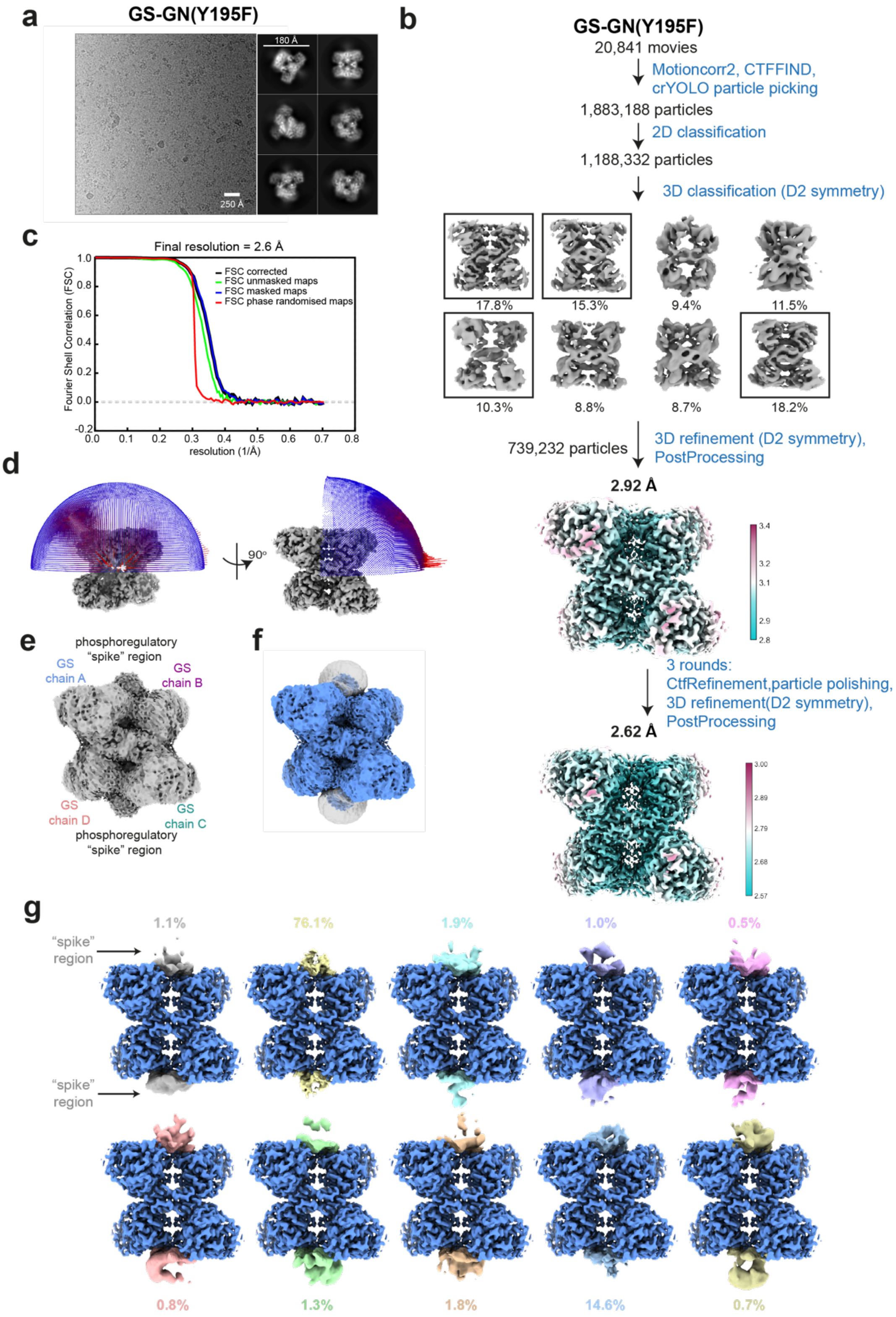
a
GS-GN(Y195F)
180 Å
250 Å
b
GS-GN(Y195F)
20,841 movies
Motioncorr2, CTFFIND, crYOLO particle picking
1,883,188 particles
2D classification
1,188,332 particles
3D classification (D2 symmetry)
17.8%
15.3%
9.4%
11.5%
10.3%
8.8%
8.7%
18.2%
739,232 particles
3D refinement (D2 symmetry), PostProcessing
2.92 Å
3.4
3.2
3.1
2.9
2.8
3 rounds: CtfRefinement,particle polishing, 3D refinement(D2 symmetry), PostProcessing
2.62 Å
3.00
2.89
2.79
2.68
2.57
c
Final resolution = 2.6 Å
FSC corrected
FSC unmasked maps
FSC masked maps
FSC phase randomised maps
Fourier Shell Correlation (FSC)
resolution (1/Å)
d
90°
e
phosphoregulatory "spike" region
GS chain A
GS chain B
GS chain D
GS chain C
phosphoregulatory "spike" region
f
g
"spike" region
"spike" region
1.1%
76.1%
1.9%
1.0%
0.5%
0.8%
1.3%
1.8%
14.6%
0.7%

Supplementary Fig. 5 Cryo-electron microscopy analysis of human GS-GN Y195F complex
a Representative micrograph (out of 20,841 micrographs collected), and a selection of 2D class averages. **b** Flow chart of data processing strategy. 3D classes boxed were selected for subsequent processing. Final map at a global resolution of 2.6 Å is coloured by local resolution. **c** FSC curve for the final map. The resolution was calculated using the gold-standard FSC cut-off at 0.143 frequency. **d** Euler angle distribution of particles used for the 3D reconstruction. Height of rods represents number of particles. **e** A 3D refinement map shown at a lower threshold to visualise the" spike" density. **f** Input map and mask used in the focussed 3D classification shown in g. **g** Focussed 3D classification of the phosphoregulatory “spike” region reveals flexibility of this inter subunit domain. 3D classes and percentage population are indicated with different colours and the 3D refined map of the core region is coloured blue.

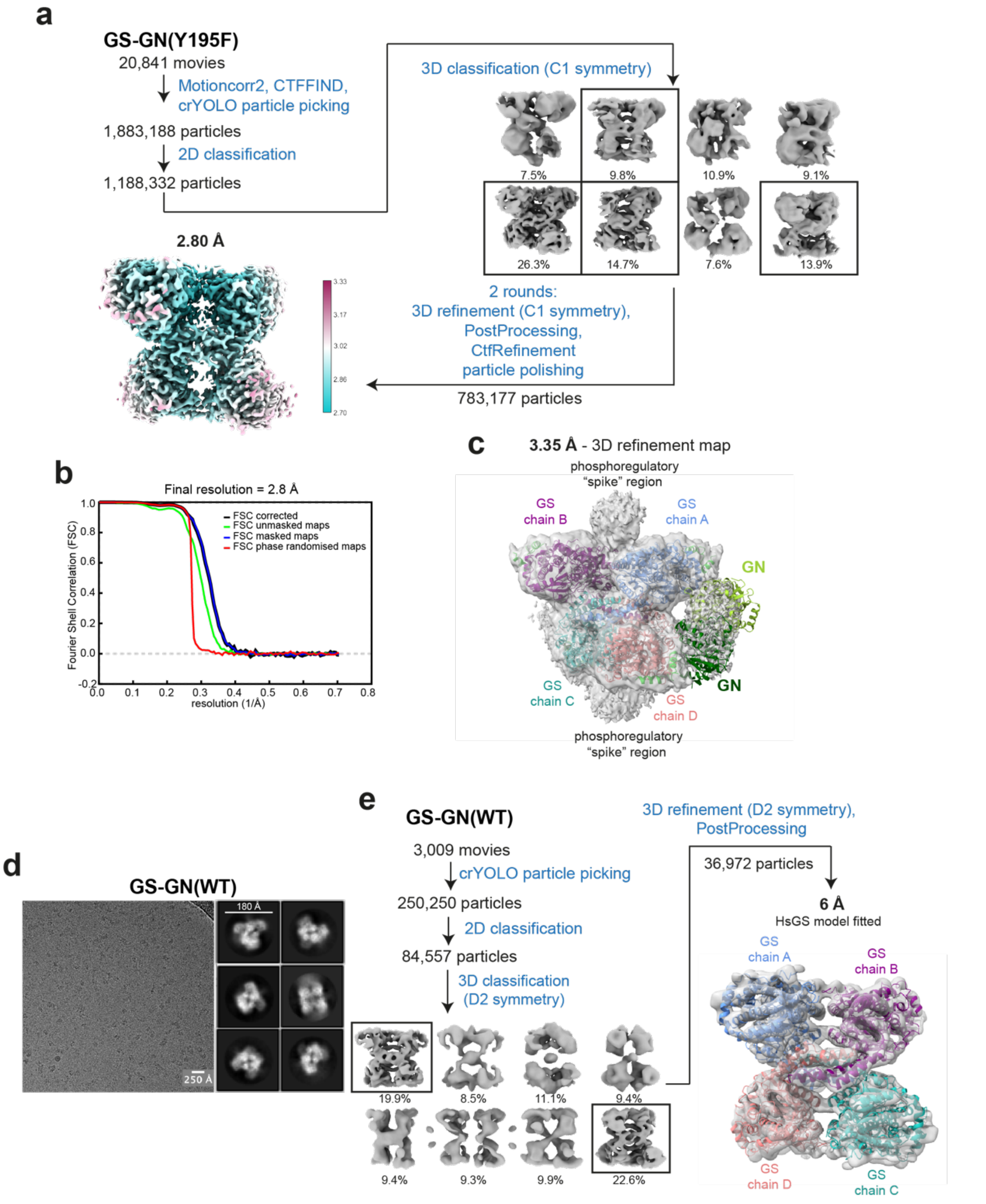

Supplementary Fig. 6 Cryo-electron microscopy analysis of human GS-GN Y195F complex without the application of symmetry averaging and cryo-electron microscopy analysis of GS-GN

a Flow chart of data processing strategy used. 3D classes boxed were selected for subsequent processing. Final map at a global resolution of 2.8 Å is shown. **b** FSC curve for the final map. The resolution was calculated using the gold-standard FSC cut-off at 0.143 frequency. **c** At a lower threshold, the 3.35 Å 3D refinement map has sufficient density for GN to allow the human GN crystal structure (PDB ID 3T7O) to be fitted. **d** Representative micrograph (out of 3,009 micrographs collected) for the GS-GN cryo-EM dataset. **e** GS-GN WT 6 Å map fits HsGS structure, showing little structural difference between the GS-GN(WT) and GS-GN(Y195F) complexes, at this resolution.

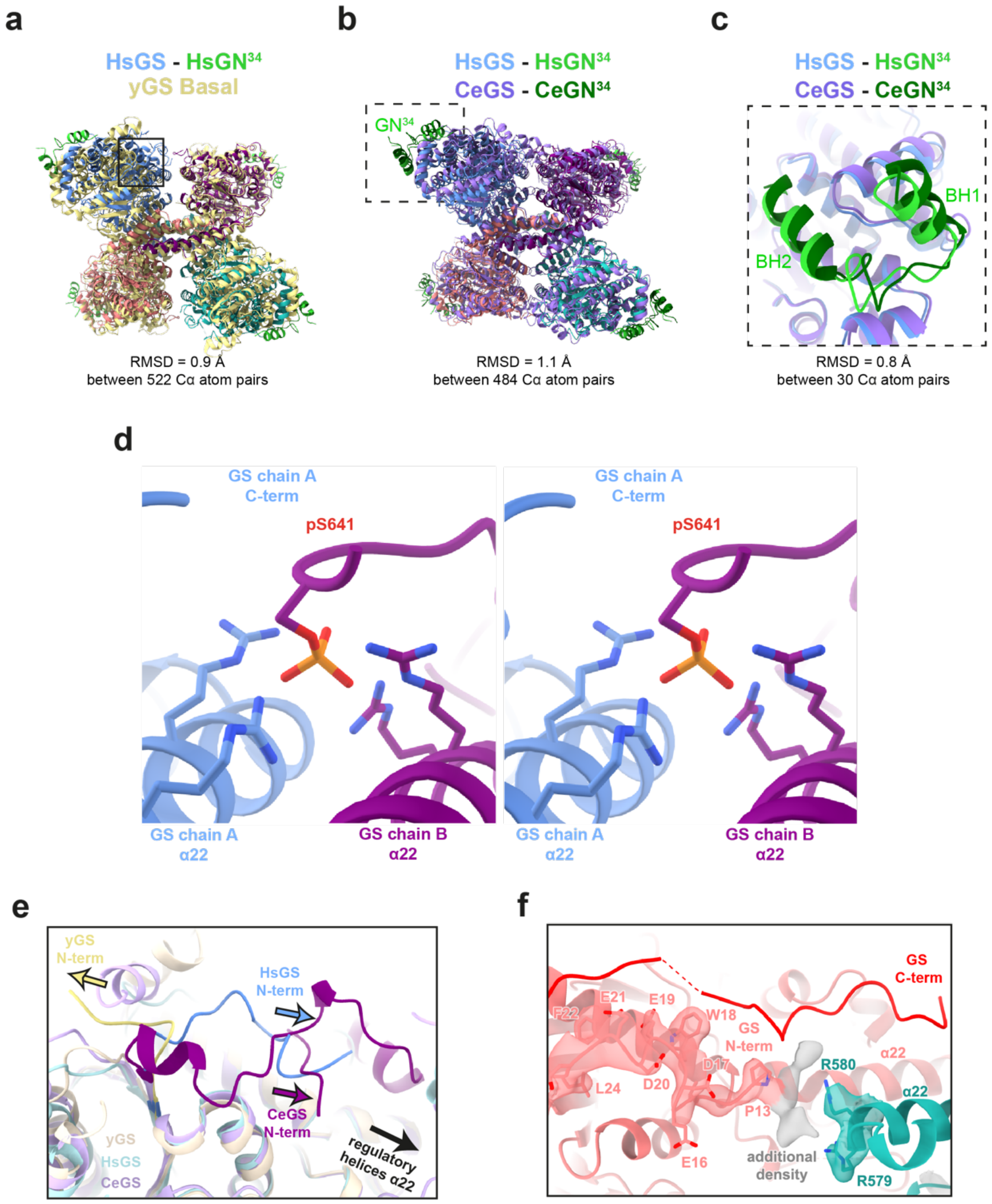

Supplementary Fig. 7 Comparison of human tetrameric GS with yeast GS and *C. elegans* GS-GN^{34} crystal structures

a Human GS has a tetrameric arrangement, agreeing with crystal structures of yeast (yellow, PDB ID 3NAZ) **b** and *C. elegans* (PDB ID 4QLB). The area boxed is shown in **c** HsGN^{34} is consistent with the CeGS-GN^{34} structure (PDB ID 4QLB). **d** Stereoview of pS641 interacting with the arginine cradle on the regulatory helices α22. **e** Human and *C. elegans* N-terminal tails are situated towards the regulatory helices. However, yeast N-terminus is pointing away from the regulatory helices. **f** Additional density for the human N-terminal tail can be seen near the regulatory helices, potentially interacting with R579 and R580 on the adjacent protomer. The LAFTER denoised map using C1 symmetry is shown.

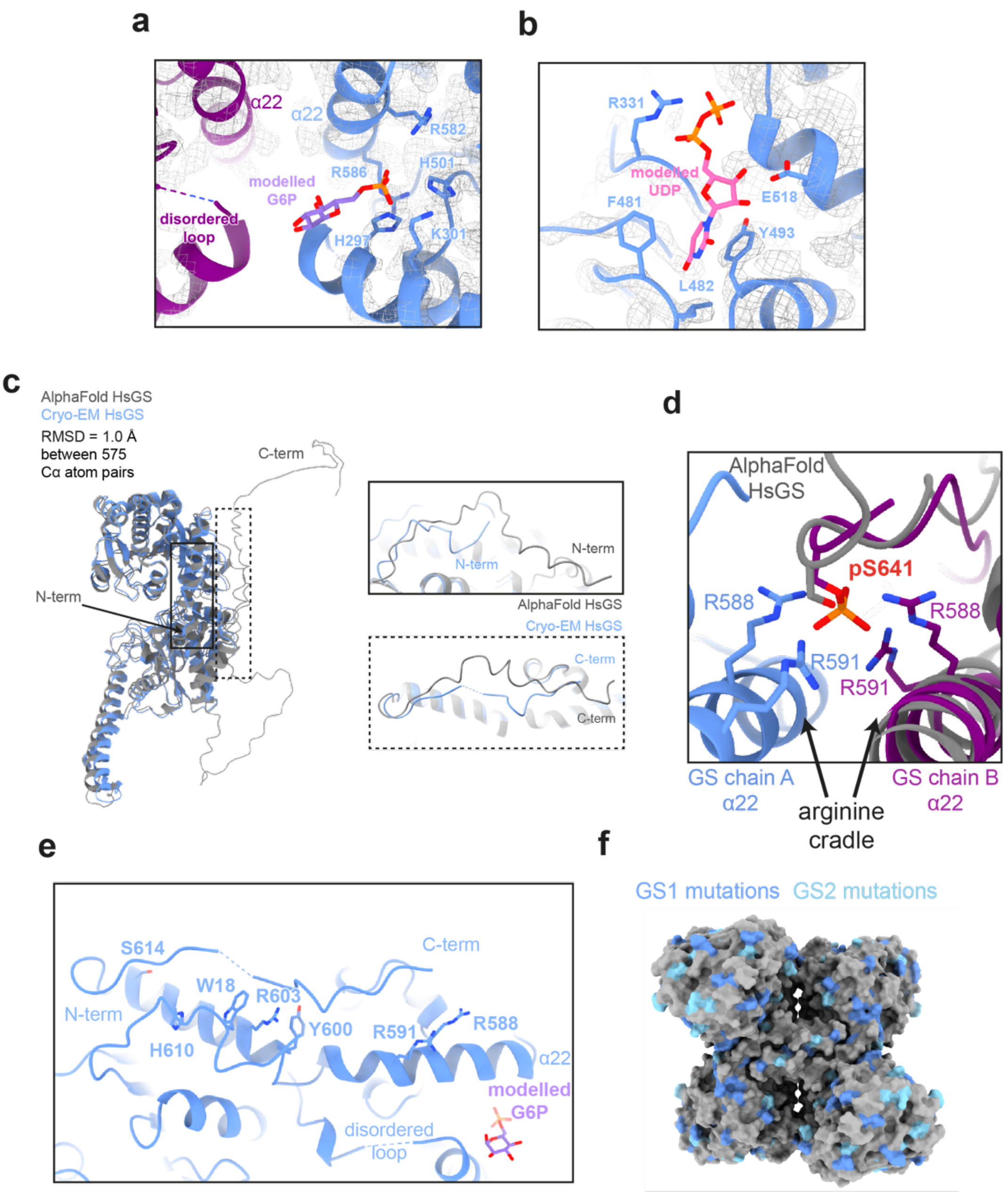

Supplementary Fig. 8 Structural analysis of inhibited human GS
a Close up view of the allosteric binding site, G6P is modelled using the yeast GS + G6P structure (PDB ID 5SUK). The residues involved in the G6P interaction are shown as sticks. **b** Close up view of the active site. UDP is modelled in the active site and the residues involved in the interaction are shown and labelled. **c** Comparison of human GS monomer model with the AlphaFold model (accessed on 1 October 2021) and position of the N- and C- terminal tails **d** Comparison of human GS pS641 model with the AlphaFold model. **e** The N- and C-terminus of one GS protomer. The residues shown are mutated in this study. G6P is modelled and the disordered loop is labelled. **f** Human GS1 and GS2 mutants mapped onto a surface representation of HsGS (reported here).

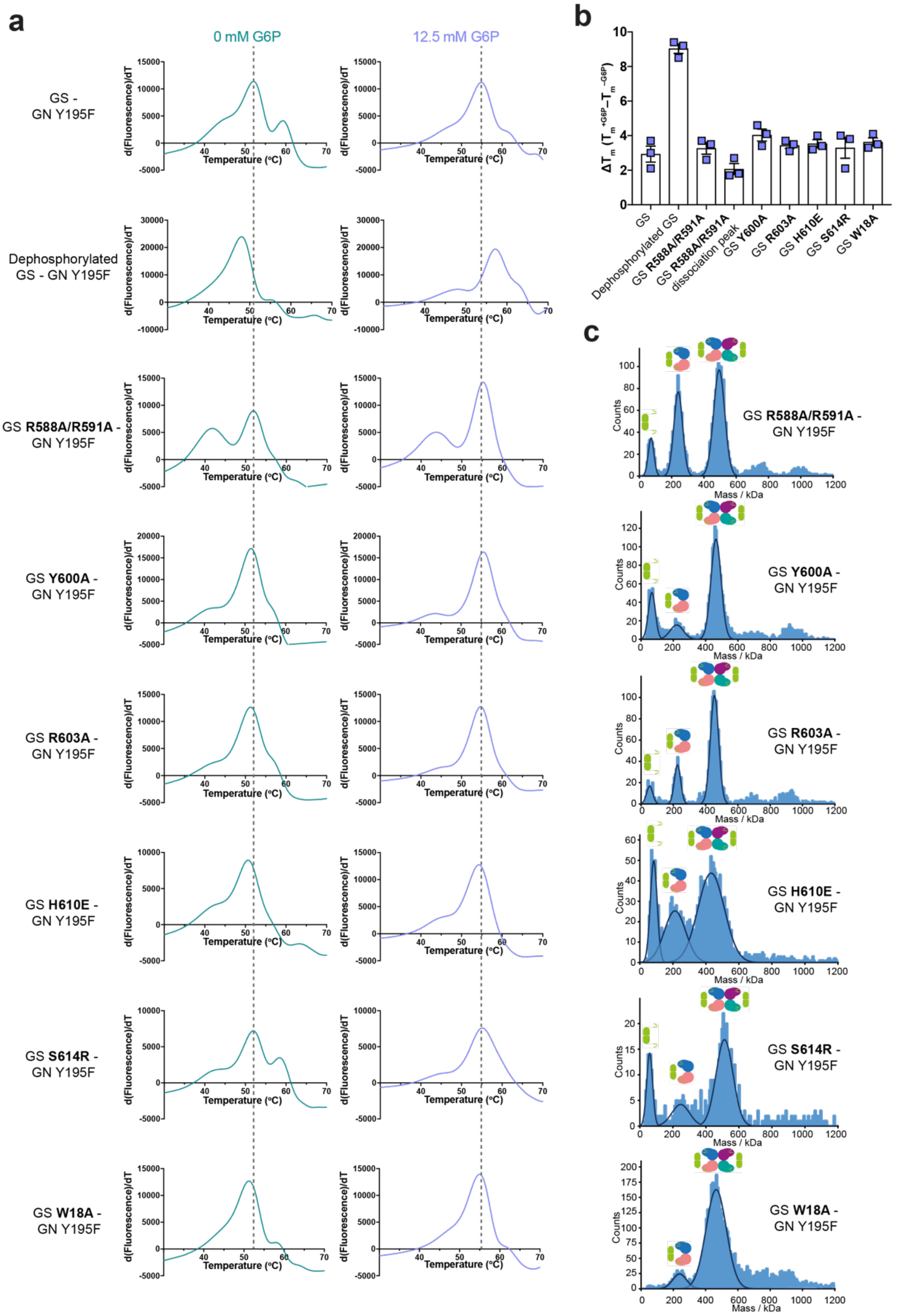

Supplementary Fig. 9 Stability of GS WT and mutants in the GS-GN(Y195F) complex
a Differential scanning fluorimetry (DSF) analysis of GS WT, various mutants and dephosphorylated GS in the GS-GN(Y195F) complex. Addition of G6P leads to stabilisation of the complex, seen by an increase in melting temperature **b** Changes in melting temperature (ΔT_m) was calculated by subtracting the T_m of 0 mM G6P of the corresponding protein. Data in **b** is shown as mean +/- S.E.M. Data in **a** and **b** are mean from three independent experiments carried out in technical duplicates (dephosphorylated GS) and triplicates (WT and mutant GS). Source data are provided as a Source Data file. **c** Mass photometry analysis of GS mutants in the GS-GN(Y195F).

Supplementary Table 1: Cryo-EM data collection, refinement and validation statistics

	**GS-GN(Y195F) PDB 7ZBN EMDB-14587**	**GS-GN**
**Data collection and processing**		
Microscope	FEI Titan KRIOS	FEI Titan KRIOS
Detector	Falcon 4	Falcon III
Energy Filter (eV)	10	/
Magnification (x)	165,000	75,000
Voltage (kV)	300	300
Electron exposure (e^-/Å2)	34.8	85
Defocus range (μm)	-1 to -2.2	-1.7 to -3.1
Pixel size (Å)	0.71	1.065
Initial particle images (no.)	1,883,188	250,250
Final particle images, D2 symmetry (no.)	739,232	36,972
Final particle images, C1 symmetry (no.)	783,177	/
Map resolution, D2 symmetry (Å)	2.62	6
Map resolution, C1 symmetry (Å)	2.8	0.143
FSC threshold	0.143	0.143
**Refinement**		
Model resolution (Å)	2.7	
FSC threshold	0.5	
Model composition		
Non-hydrogen atoms	41588	
Protein residues	2608	
Ligands	0	
B factors ($Å^2$)		
Protein (min/max/mean)	5.81/46.06/19.42	
Map-model CC		
CC (mask)	0.81	
CC (volume)	0.76	
CC (peaks)	0.69	
CC (box)	0.71	
R.m.s. deviations		
Bond lengths (Å)	0.006	
Bond angle (°)	0.551	
**Validation**		
Molprobity score	1.71	
Clashscore	10.45	
Rotamers outliers (%)	0	
Ramachandran plot		
Favored (%)	97.01	
Allowed (%)	2.99	
Outliers (%)	0	

Supplementary Table 2: Mutations of GS1 and GS2 genes. Source: clinvar, last accessed 4 October 2021

**GS1 mutations**					
**CDS mutation**	**AA mutation**	**Type**	**Accession code (clinvar)**	**Condition(s)**	**Residue interaction**
c.1A>C	M1L	missense	VCV000430320	Glycogen storage disease 0, muscle	
c.3G>T	M1I	missense	VCV001039847	Glycogen storage disease 0, muscle	
c.20T>C	L7S	missense	VCV000999389	Glycogen storage disease 0, muscle	
c.40G>C	G14R	missense	VCV000964057	Glycogen storage disease 0, muscle	
c.56A>T	E19V	missense	VCV001063670	not provided	
c.80C>T	A27V	missense	VCV001064089	Glycogen storage disease 0, muscle	
c.101G>T	W34L	missense	VCV000429776	Glycogen storage disease 0, muscle	
c.114C>G	N38K	missense	VCV000946451	Glycogen storage disease 0, muscle	
c.134C>T	T45M	missense	VCV000945011	Glycogen storage disease 0, muscle	
c.178G>A	D60N	missense	VCV001002555	Glycogen storage disease 0, muscle	
c.213G>C	Q71H	missense	VCV000642436	not provided	
c.214G>T	G72C	missense	VCV001014411	Glycogen storage disease 0, muscle	
c.217G>A	V73M	missense	VCV000970915	Glycogen storage disease 0, muscle	
c.221G>C	R74T	missense	VCV000645191	Glycogen storage disease 0, muscle	
c.254C>T	P85L	missense	VCV000960357	Glycogen storage disease 0, muscle	
c.278C>T	S93F	missense	VCV000854343	Glycogen storage disease 0, muscle	
c.310G>A	G104R	missense	VCV000450024	not provided	
c.314G>A	R105H	missense	VCV000654802	Glycogen storage disease 0, muscle	
c.324C>G	I108M	missense	VCV000998960	Glycogen storage disease 0, muscle	
c.362C>T	A121V	missense	VCV001040534	Glycogen storage disease 0, muscle	
c.395A>G	E132G	missense	VCV000567434	Glycogen storage disease 0, muscle	
c.418G>A	G140R	missense	VCV001004862	Glycogen storage disease 0, muscle	GN[34] interaction
c.425C>T	P142L	missense	VCV000941325	Glycogen storage disease 0, muscle	GN[34] interaction
c.448G>A	D150N	missense	VCV000942370	Glycogen storage disease 0, muscle	
c.500C>A	A167E	missense	VCV000652496	Glycogen storage disease 0, muscle	
c.505A>G	S169G	missense	VCV000894589	Glycogen storage disease 0, muscle	

c.524T>C	V175A	missense	VCV000946427	Glycogen storage disease 0, muscle	
c.553G>A	G185S	missense	VCV000862647	Glycogen storage disease 0, muscle	
c.556G>A	V186I	missense	VCV000329823	Glycogen storage disease 0, muscle	
c.578C>T	A193V	missense	VCV000847954	Glycogen storage disease 0, muscle	GN[34] interaction
c.584G>A	R195Q	missense	VCV000967367	Glycogen storage disease 0, muscle	GN[34] interaction
c.631C>T	R211C	missense	VCV000970295	Glycogen storage disease 0, muscle	
c.646G>A	G216S	missense	VCV000939691	Glycogen storage disease 0, muscle	
c.646G>T	G216C	missense	VCV000966878	Glycogen storage disease 0, muscle	
c.650C>T	A217V	missense	VCV000949974	Glycogen storage disease 0, muscle	
c.652G>A	V218M	missense	VCV000835130	Glycogen storage disease 0, muscle	
c.652G>C	V218L	missense	VCV001054282	Glycogen storage disease 0, muscle	
c.666C>G	N222K	missense	VCV000836150	Glycogen storage disease 0, muscle	sugar binding
c.666C>A	N222K	missense	VCV001037659	Glycogen storage disease 0, muscle	sugar binding
c.683A>G	N228S	missense	VCV001007054	Glycogen storage disease 0, muscle	GN[34] interaction
c.704A>T	E235V	missense	VCV000856687	Glycogen storage disease 0, muscle	
c.722G>A	R241Q	missense	VCV001018886	Glycogen storage disease 0, muscle	
c.754G>A	A252T	missense	VCV000391854	Glycogen storage disease 0, muscle	
c.760G>A	V254I	missense	VCV000940671	Glycogen storage disease 0, muscle	
c.793G>A	E265K	missense	VCV000653921	Glycogen storage disease 0, muscle	
c.803A>T	H268L	missense	VCV000894180	Glycogen storage disease 0, muscle	
c.872A>G	H291R	missense	VCV001023691	Glycogen storage disease 0, muscle	G6P binding
c.944A>T	H315L	missense	VCV001015336	Glycogen storage disease 0, muscle	
c.1012G>C	G338R	missense	VCV000945364	Glycogen storage disease 0, muscle	
c.1015G>T	A339S	missense	VCV001013781	Glycogen storage disease 0, muscle	
c.1020C>A	D340E	missense	VCV000955642	Glycogen storage disease 0, muscle	
c.1021G>A	V341I	missense	VCV000329819	Glycogen storage disease 0, muscle	
c.1049A>G	N350S	missense	VCV001024950	Glycogen storage disease 0, muscle	
c.1052A>G	Y351C	missense	VCV000659273	Glycogen storage disease 0, muscle	sugar binding
c.1075G>A	E359K	missense	VCV001056519	Glycogen storage disease 0, muscle	
c.1077_1078delinsAA	Q360K	missense	VCV000858591	Glycogen storage disease 0, muscle	

c.1078C>A	Q360K	missense	VCV000894178	Glycogen storage disease 0, muscle	
c.1112G>A	R371Q	missense	VCV000844943	Glycogen storage disease 0, muscle	
c.1121A>G	N374S	missense	VCV000585159	Glycogen storage disease 0, muscle	
c.1127A>G	N376S	missense	VCV001007393	Glycogen storage disease 0, muscle	sugar binding
c.1144G>A	G382S	missense	VCV000944904	Glycogen storage disease 0, muscle	
c.1145G>A	G382D	missense	VCV000329816	Glycogen storage disease 0, muscle	tetramerization domain
c.1156C>T	R386C	missense	VCV000893350	Glycogen storage disease 0, muscle	tetramerization domain
c.1184C>T	T395M	missense	VCV000893348	Glycogen storage disease 0, muscle	tetramerization domain
c.1190A>G	K397R	missense	VCV000641050	Glycogen storage disease 0, muscle	tetramerization domain
c.1236C>G	S412R	missense	VCV000662709	Glycogen storage disease 0, muscle	phosphorylation site (identified by phosphorylation mapping)
c.1243G>A	D415N	missense	VCV000808611	Glycogen storage disease 0, muscle	
c.1246A>G	M416V	missense	VCV000329814	Glycogen storage disease 0, muscle	tetramerization domain
c.1279A>G	M427V	missense	VCV001017549	Glycogen storage disease 0, muscle	
c.1303A>G	T435A	missense	VCV000893132	Glycogen storage disease 0, muscle	
c.1315_1316delinsAA	S439N	missense	VCV000649510	Glycogen storage disease 0, muscle	
c.1318T>C	F440L	missense	VCV000498163	not provided	sugar binding
c.1324C>G	P442A	missense	VCV000498167	Glycogen storage disease 0, muscle	sugar binding
c.1324C>A	P442T	missense	VCV000936512	Glycogen storage disease 0, muscle	sugar binding
c.1363C>T	P455S	missense	VCV001004987	Glycogen storage disease 0, muscle	
c.1407T>A	S469R	missense	VCV000893129	Glycogen storage disease 0, muscle	
c.1412A>G	D471G	missense	VCV000947297	Glycogen storage disease 0, muscle	
c.1432C>T	H478Y	missense	VCV000942790	Glycogen storage disease 0, muscle	
c.1436C>T	P479L	missense	VCV000643030	Glycogen storage disease 0, muscle	
c.1475A>T	D492V	missense	VCV000938769	Glycogen storage disease 0, muscle	
c.1492C>T	R498C	missense	VCV001014236	Glycogen storage disease 0, muscle	
c.1520C>T	S507F	missense	VCV000546850	not provided	
c.1559C>T	T520M	missense	VCV001026554	Glycogen storage disease 0, muscle	
c.1615G>A	E539K	missense	VCV000329810	Neuroferritinopathy, Hereditary hyperferritinemia with congenital cataracts, Glycogen storage disease 0, muscle	sugar binding

c.1754A>G	Q585R	missense	VCV000859569	Glycogen storage disease 0, muscle	regulatory helix, α22
c.1822G>A	A608T	missense	VCV001016164	Glycogen storage disease 0, muscle	
c.1823C>G	A608G	missense	VCV001052744	Glycogen storage disease 0, muscle	
c.1834G>C	A612P	missense	VCV001051757	Glycogen storage disease 0, muscle	
c.1835C>T	A612V	missense	VCV001061438	Glycogen storage disease 0, muscle	
c.1859A>G	H620R	missense	VCV001043805	Glycogen storage disease 0, muscle	
c.1879G>A	E627K	missense	VCV000576955	Glycogen storage disease 0, muscle	
c.1898G>T	G633V	missense	VCV001021949	Glycogen storage disease 0, muscle	
c.1909C>G	P637A	missense	VCV000651479	Glycogen storage disease 0, muscle	
c.1922C>T	S641L	missense	VCV000916245	Glycogen storage disease 0, muscle	phosphorylation site
c.1961C>T	P654L	missense	VCV000329807	Glycogen storage disease 0, muscle	
c.1985A>G	D662G	missense	VCV000934446	Glycogen storage disease 0, muscle	
c.1990C>T	R664W	missense	VCV000329806	Glycogen storage disease 0, muscle	
c.2008G>A	E670K	missense	VCV001009397	Glycogen storage disease 0, muscle	
c.2009A>G	E670G	missense	VCV000999215	Glycogen storage disease 0, muscle	
c.2014G>A	G672S	missense	VCV000945494	Glycogen storage disease 0, muscle	
c.2017G>A	E673K	missense	VCV000957665	Glycogen storage disease 0, muscle	
c.2021G>A	R674H	missense	VCV001021262	Glycogen storage disease 0, muscle	
c.2032G>A	D678N	missense	VCV001024883	Glycogen storage disease 0, muscle	
c.2035G>A	E679K	missense	VCV000894150	Glycogen storage disease 0, muscle	
c.2042C>T	A681V	missense	VCV000985784	Inborn genetic diseases	
c.2044G>A	A682T	missense	VCV001018906	Glycogen storage disease 0, muscle	
c.2065C>T	R689C	missense	VCV001006088	Glycogen storage disease 0, muscle	
c.2096G>T	C699F	missense	VCV001010299	Glycogen storage disease 0, muscle	
c.2105C>T	S702F	missense	VCV000836504	Glycogen storage disease 0, muscle	
c.2120A>C	K707T	missense	VCV000894149	Glycogen storage disease 0, muscle	
c.2122C>G	R708G	missense	VCV000844469	Glycogen storage disease 0, muscle	
c.2206C>T	R736C	missense	VCV000837003	Glycogen storage disease 0, muscle	
c.2207G>A	R736H	missense	VCV000329804	Glycogen storage disease 0, muscle, Neuroferritinopathy, Hereditary hyperferritinemia with congenital cataracts	

c.907C>T	R303*	nonsense	VCV000965769	Glycogen storage disease 0, muscle	
c.913C>T	Q305*	nonsense	VCV001074189	Glycogen storage disease 0, muscle	
c.1384C>T	R462*	nonsense	VCV000016057	Glycogen storage disease 0, muscle	sugar binding
c.1615G>T	E539*	nonsense	VCV000570876	Glycogen storage disease 0, muscle	sugar binding
c.160dup	T54fs	frameshift - duplication	VCV000422359	not provided	
c.162_163del	D56fs	frameshift - deletion	VCV000128236	Glycogen storage disease 0, muscle	
c.699_700del	R236fs	frameshift - deletion	VCV001069149	Glycogen storage disease 0, muscle	
c.1204del	R402fs	frameshift - deletion	VCV000567037	Glycogen storage disease 0, muscle	
c.2207del	R736fs	frameshift - deletion	VCV000861694	Glycogen storage disease 0, muscle	
**GS2 mutations**					
**CDS mutation**	**AA mutation**	**Type**	**Accession code (clinvar)**	**Condition(s)**	**Residue interaction**
c.50A>G	Q17R	missense	VCV000665936	Glycogen storage disease due to hepatic glycogen synthase deficiency	
c.116A>G	N39S	missense	VCV000016054	Glycogen storage disease due to hepatic glycogen synthase deficiency	
c.154G>A	A52T	missense	VCV000308011	Glycogen storage disease due to hepatic glycogen synthase deficiency	
c.215A>G	H72R	missense	VCV000884167	Glycogen storage disease due to hepatic glycogen synthase deficiency	
c.279C>G	D93E	missense	VCV000214522	not specified	
c.280G>A	A94T	missense	VCV000137522	Glycogen storage disease due to hepatic glycogen synthase deficiency	
c.289A>G	K97E	missense	VCV000308009	Glycogen storage disease due to hepatic glycogen synthase deficiency	sugar binding
c.299G>A	C100Y	missense	VCV000214527	not provided	
c.395G>A	G132D	missense	VCV000214523	not specified	
c.421G>A	G141S	missense	VCV000137523	Glycogen storage disease due to hepatic glycogen synthase deficiency	
c.427C>T	P143S	missense	VCV000861394	Glycogen storage disease due to hepatic glycogen synthase deficiency	GN[34] interaction
c.470C>T	S157F	missense	VCV000999043	Glycogen storage disease due to hepatic glycogen synthase deficiency	
c.520T>C	Y174H	missense	VCV000380333	not specified	
c.526G>A	V176I	missense	VCV000736468	Glycogen storage disease due to hepatic glycogen synthase deficiency	
c.556A>T	I186F	missense	VCV000214524	not specified	
c.577G>A	A193T	missense	VCV000261471	Glycogen storage disease due to hepatic glycogen synthase deficiency	GN[34] interaction

c.653T>C	I218T	missense	VCV000964160	Glycogen storage disease due to hepatic glycogen synthase deficiency	
c.753C>G	C251W	missense	VCV001036478	Glycogen storage disease due to hepatic glycogen synthase deficiency	GN[34] interaction
c.755C>G	A252G	missense	VCV000308003	Glycogen storage disease due to hepatic glycogen synthase deficiency	
c.799G>A	E267K	missense	VCV000882213	Glycogen storage disease due to hepatic glycogen synthase deficiency	
c.956A>T	D319V	missense	VCV000376836	not provided	
c.1015G>C	A339P	missense	VCV000016052	Glycogen storage disease due to hepatic glycogen synthase deficiency	
c.1087A>G	M363V	missense	VCV000261462	Glycogen storage disease due to hepatic glycogen synthase deficiency	
c.1129G>A	V377M	missense	VCV000880824	Glycogen storage disease due to hepatic glycogen synthase deficiency	
c.1171G>T	D391Y	missense	VCV000884120	Glycogen storage disease due to hepatic glycogen synthase deficiency	tetramerization domain
c.1245C>G	D415E	missense	VCV000137524	Glycogen storage disease due to hepatic glycogen synthase deficiency	tetramerization domain
c.1277C>T	T426I	missense	VCV000214531	not provided	tetramerization domain
c.1279A>C	I427L	missense	VCV001058720	Glycogen storage disease due to hepatic glycogen synthase deficiency	
c.1334C>T	T445M	missense	VCV000952317	Glycogen storage disease due to hepatic glycogen synthase deficiency	
c.1336C>G	H446D	missense	VCV000016056	Glycogen storage disease due to hepatic glycogen synthase deficiency	
c.1418T>G	V473G	missense	VCV000307993	Glycogen storage disease due to hepatic glycogen synthase deficiency	
c.1427T>A	I476N	missense	VCV000214532	not provided	
c.1436C>A	P479Q	missense	VCV000016051	Glycogen storage disease due to hepatic glycogen synthase deficiency	
c.1447T>C	S483P	missense	VCV000016055	Glycogen storage disease due to hepatic glycogen synthase deficiency	
c.1472T>G	M491R	missense	VCV000016053	Glycogen storage disease due to hepatic glycogen synthase deficiency	
c.1475A>T	D492V	missense	VCV000418239	not provided	
c.1477T>A	Y493N	missense	VCV000521873	Inborn genetic diseases	active site
c.1522T>G	Y508D	missense	VCV000418240	not provided	sugar binding
c.1549G>C	A517P	missense	VCV000307992	Glycogen storage disease due to hepatic glycogen synthase deficiency	
c.1553A>C	E518A	missense	VCV000214533	Glycogen storage disease due to hepatic glycogen synthase deficiency	active site
c.1636A>G	T546A	missense	VCV000137526	Glycogen storage disease due to hepatic glycogen synthase deficiency	
c.1657G>A	V553I	missense	VCV000214525	not specified	

c.1672C>T	R558C	missense	VCV000648650	Glycogen storage disease due to hepatic glycogen synthase deficiency	sugar binding
c.1745G>A	R582K	missense	VCV000534624	Glycogen storage disease due to hepatic glycogen synthase deficiency	G6P binding
c.1774C>G	L592V	missense	VCV000653240	Glycogen storage disease due to hepatic glycogen synthase deficiency	regulatory helix, α22
c.1789G>A	D597N	missense	VCV000214535	not provided	
c.1790A>G	D597G	missense	VCV000307990	Glycogen storage disease due to hepatic glycogen synthase deficiency	
c.1820A>G	H607R	missense	VCV000307989	Glycogen storage disease due to hepatic glycogen synthase deficiency	
c.1829A>G	H610R	missense	VCV000936980	Glycogen storage disease due to hepatic glycogen synthase deficiency	
c.1880C>T	S627T	missense	VCV000766741	Glycogen storage disease due to hepatic glycogen synthase deficiency	
c.1889C>T	T630M	missense	VCV000719047	Glycogen storage disease due to hepatic glycogen synthase deficiency	
c.1906T>C	Y636H	missense	VCV000658693	Glycogen storage disease due to hepatic glycogen synthase deficiency	
c.1965G>C	Q655H	missense	VCV000137528	Glycogen storage disease due to hepatic glycogen synthase deficiency	
c.2005G>A	D669N	missense	VCV000214526	Glycogen storage disease due to hepatic glycogen synthase deficiency	
c.2054T>C	F685S	missense	VCV000137529	Glycogen storage disease due to hepatic glycogen synthase deficiency, not provided	
c.2068G>T	V690F	missense	VCV000720229	not provided	
c.2072C>T	P691L	missense	VCV000798247	Glycogen storage disease due to hepatic glycogen synthase deficiency	
c.547C>T	Q183*	nonsense	VCV000214529	Glycogen storage disease due to hepatic glycogen synthase deficiency	
c.574C>T	R192*	nonsense	VCV000214530	Glycogen storage disease due to hepatic glycogen synthase deficiency	GN[34] interaction
c.736C>T	R246*	nonsense	VCV000016049	Glycogen storage disease due to hepatic glycogen synthase deficiency	GN[34] interaction
c.925C>T	R309*	nonsense	VCV000078954	Glycogen storage disease due to hepatic glycogen synthase deficiency	
c.1156C>T	R386*	nonsense	VCV000569452	Glycogen storage disease due to hepatic glycogen synthase deficiency	
c.465del	F155fs	frameshift - deletion	VCV000937499	Glycogen storage disease due to hepatic glycogen synthase deficiency	
c.1081del	T361fs	frameshift - deletion	VCV000667422	Glycogen storage disease	
c.1974dup	V659fs	frameshift - duplication	VCV000631679	Glycogen storage disease due to hepatic glycogen synthase deficiency	
