# Supplementary material for: Mechanism of glycogen synthase inactivation and interaction with glycogenin
Source: Nat Commun. 2022 Jun 11;13:3372. doi: 10.1038/s41467-022-31109-6 (PMC9188544; doi:10.1038/s41467-022-31109-6)
Supplement: Supplementary file 3 — Description of Additional Supplementary Files [file 41467_2022_31109_MOESM3_ESM.pdf]

File name: Supplementary Data 1

Description: All data from protein identification mass spectrometry analysis.

File name: Supplementary Movie 1

Description: Movie showing one component from 3D variability analysis, showing slight flexing of the GS tetramer.
