# Supplementary material for: Mechanism of glycogen synthase inactivation and interaction with glycogenin
Source: Nat Commun. 2022 Jun 11;13:3372. doi: 10.1038/s41467-022-31109-6 (PMC9188544; doi:10.1038/s41467-022-31109-6)
Supplement: Supplementary file 6 — Reporting Summary [file 41467_2022_31109_MOESM6_ESM.pdf]

nature portfolio

Corresponding author(s): Elton Zeqiraj

Last updated by author(s): 19/May/2022

Reporting Summary

Nature Portfolio wishes to improve the reproducibility of the work that we publish. This form provides structure for consistency and transparency in reporting. For further information on Nature Portfolio policies, see our Editorial Policies and the Editorial Policy Checklist.

Statistics

For all statistical analyses, confirm that the following items are present in the figure legend, table legend, main text, or Methods section.

n/a	Confirmed	
☐	☒	The exact sample size (*n*) for each experimental group/condition, given as a discrete number and unit of measurement
☐	☒	A statement on whether measurements were taken from distinct samples or whether the same sample was measured repeatedly
☐	☒	The statistical test(s) used AND whether they are one- or two-sided<br>*Only common tests should be described solely by name; describe more complex techniques in the Methods section.*
☒	☐	A description of all covariates tested
☒	☐	A description of any assumptions or corrections, such as tests of normality and adjustment for multiple comparisons
☐	☒	A full description of the statistical parameters including central tendency (e.g. means) or other basic estimates (e.g. regression coefficient) AND variation (e.g. standard deviation) or associated estimates of uncertainty (e.g. confidence intervals)
☐	☒	For null hypothesis testing, the test statistic (e.g. *F*, *t*, *r*) with confidence intervals, effect sizes, degrees of freedom and *P* value noted<br>*Give P values as exact values whenever suitable.*
☒	☐	For Bayesian analysis, information on the choice of priors and Markov chain Monte Carlo settings
☒	☐	For hierarchical and complex designs, identification of the appropriate level for tests and full reporting of outcomes
☒	☐	Estimates of effect sizes (e.g. Cohen's *d*, Pearson's *r*), indicating how they were calculated

Our web collection on statistics for biologists contains articles on many of the points above.

Software and code

Policy information about availability of computer code

Data collection	Cryo-EM data were collected using ThermoFisher EPU software (v2.13). Negative stain EM data were collected using ThermoFisher TEM imaging and analysis (TIA).
Data analysis	Cryo-EM data and models were analyzed using: Relion (3.1.1), cryoSPARC (3.2.0), crYOLO (1.3.5), MotionCorr2 (1.2.1), gCTF (1.18), CTFFINF (v4.1), Coot (0.9.2-pre), Phenix (1.19), Chimera (1.12) and ChimeraX (1.3.0). Statistical analysis was performed using GraphPad Prism (v9.3.1). Mass spectrometry data was analyzed by Proteome Discoverer (v1.4).

For manuscripts utilizing custom algorithms or software that are central to the research but not yet described in published literature, software must be made available to editors and reviewers. We strongly encourage code deposition in a community repository (e.g. GitHub). See the Nature Portfolio guidelines for submitting code & software for further information.

Data

Policy information about availability of data

All manuscripts must include a data availability statement. This statement should provide the following information, where applicable:

- Accession codes, unique identifiers, or web links for publicly available datasets
- A description of any restrictions on data availability
- For clinical datasets or third party data, please ensure that the statement adheres to our policy

The cryo-EM maps have been deposited in the Electron Microscopy Data Bank under the accession code EMDB-14587. Coordinates have been deposited in the Protein Data Bank under the accession code 7ZBN. The source data underlying Fig. 1d, 2a, 2b, 4a, 4b, 4c, 4d, 4e and Supplementary Fig. 3a-e, 4a, 4c, and 9b are provided as a Source Data file. Mass spectrometry data was analyzed using the UniProt Human reviewed database (updated April 2020) (https://www.uniprot.org/).

Field-specific reporting

Please select the one below that is the best fit for your research. If you are not sure, read the appropriate sections before making your selection.

☒ Life sciences ☐ Behavioural & social sciences ☐ Ecological, evolutionary & environmental sciences

For a reference copy of the document with all sections, see nature.com/documents/nr-reporting-summary-flat.pdf

Life sciences study design

All studies must disclose on these points even when the disclosure is negative.

Sample size	Cryo-EM datasets were collected for a total of approximately 3 days of instrument time. Sample size for cryo-EM analysis was arbitrary chosen depending on sample and instrument availability and the number of particles sufficient to obtain a structure at the reported resolution.
Data exclusions	No data exclusion.
Replication	All data was replicated at least two times successfully and in line with accepted scientific standards.
Randomization	No randomization during allocation into experimental groups was performed. Sample selection bias and/or accidental bias was addressed by use of appropriate controls (e.g. use of loss-of-function mutants, presence of ligands with known / expected effect, etc.).
Blinding	Structure determinations were performed without blinding. The sample identity was known to the investigators as this was required for appropriate interpretation of EM maps and models.

Reporting for specific materials, systems and methods

We require information from authors about some types of materials, experimental systems and methods used in many studies. Here, indicate whether each material, system or method listed is relevant to your study. If you are not sure if a list item applies to your research, read the appropriate section before selecting a response.

Materials & experimental systems

n/a	Involved in the study	
☐	☒	Antibodies
☐	☒	Eukaryotic cell lines
☒	☐	Palaeontology and archaeology
☐	☒	Animals and other organisms
☒	☐	Human research participants
☒	☐	Clinical data
☒	☐	Dual use research of concern

Methods

n/a	Involved in the study	
☒	☐	ChIP-seq
☒	☐	Flow cytometry
☒	☐	MRI-based neuroimaging

Antibodies

Antibodies used

1. GS (#3893, 4) Cell Signaling Technologies (1:1000 in 5% BSA solution)
2. pGS S641 (D4H1B) (#47043, 1) Cell Signaling Technologies (1:1000 in 5% BSA solution)
3. pGS S641/S645 (07-817, 3101536) Merck Millipore (1:1000 in 5% BSA solution)
4. GN (S197C, 3rd bleed) MRC-PPU Reagents and Services (1 μg/mL in 5% BSA solution)
5. pGS S8* (YZ7516, 1st cycle, 10211) YenZym (custom generated) (0.5 μg/mL in 5% BSA solution)
6. Affinipure Donkey Anti-Rabbit IgG (H+L) HRP (711-035-152, 54959) BioRad (1:10000 in 3% NFDM)
7. Affinipure Donkey Anti-Sheep IgG (H+L) HRP (713-035-147, 147940) BioRad (1:10000 in 3% NFDM)

*Antigen peptide information for pGS S8 (pSer8, Human residues 2-14 in combination with pSer8 Mouse residues 2-14)
Human: PLNRTL-pS-MSSLPG-Ahx-C-amide; Mouse: PLSRSL-pS-VSSLPG-Ahx-C-amide

Validation

Validation of all the commercially purchased antibodies can be found on their respective websites.

1. https://www.cellsignal.com/products/primary-antibodies/glycogen-synthase-antibody/3893
2. https://www.cellsignal.com/products/primary-antibodies/phospho-glycogen-synthase-ser641-d4h1b-xp-rabbit-mab/47043?site-search-type=Products&N=4294956287&Ntt=glycogen+synthase&fromPage=plp
3. https://www.merckmillipore.com/DK/en/product/Anti-phospho-Glycogen-SynthaseSer641-Ser645-Antibody,MM_NF-07-817?ReferrerURL=https%3A%2F%2Fwww.google.com%2F&bd=1#anchor_COA
4. https://mrcppureagents.dundee.ac.uk/reagents-view-antibodies/587996

Validation for the custom generated pGS S8 antibody is shown in Supplementary Fig. 4c.

Eukaryotic cell lines

Policy information about cell lines

Cell line source(s)	Sf9 and tni cells were used for protein expression were obtained from Invitrogen (Thermofisher). HEK293FT cell line was purchased from ATCC (ATCC-CRL-1573. 293; Embryonic Kidney, Human (Homo sapiens)).
Authentication	No authentication of Sf9 or Tni cell line authentication was performed in our laboratory. HEK293FT authentication was performed by ATCC.
Mycoplasma contamination	Regular test are held in our laboratory (every 2 months). No mycoplasma contamination was detected.
Commonly misidentified lines (See ICLAC register)	Commonly misidentified lines were not used.

Animals and other organisms

Policy information about studies involving animals; ARRIVE guidelines recommended for reporting animal research

Laboratory animals	N/A
Wild animals	N/A
Field-collected samples	N/A
Ethics oversight	N/A

Note that full information on the approval of the study protocol must also be provided in the manuscript.
